# Gut Microbiota in a Viral Model of Multiple Sclerosis: Modulation and Pitfalls by Oral Antibiotic Treatment

**DOI:** 10.3390/cells14120871

**Published:** 2025-06-09

**Authors:** Ijaz Ahmad, Seiichi Omura, Sundar Khadka, Fumitaka Sato, Ah-Mee Park, Sandesh Rimal, Ikuo Tsunoda

**Affiliations:** 1 Department of Microbiology, Faculty of Medicine, Kindai University, 377-2 Ohnohigashi, Osakasayma 589-8511, Osaka, Japan; ijazahmad383@gmail.com (I.A.); somura@med.kindai.ac.jp (S.O.); cls.sundar@gmail.com (S.K.); fsato@med.kindai.ac.jp (F.S.); ampk@med.kindai.ac.jp (A.-M.P.); niensandesh@gmail.com (S.R.); 2 Department of Medicine, Duke University, Durham, NC 27708, USA; 3 Department of Arts and Science, Faculty of Medicine, Kindai University, Osakasayma 589-8511, Osaka, Japan

**Keywords:** animal models, antibody isotype, CNS demyelinating disease, intestinal bacterial flora, microbiome, neuroinflammation, paraffin section, *Picornaviridae* infections, 16S rRNA, Theiler’s virus model

## Abstract

Viral infections have been associated with multiple sclerosis (MS), an immune-mediated disease in the central nervous system (CNS). Since Theiler’s murine encephalomyelitis virus (TMEV) can induce MS-like demyelination, TMEV infection is the most widely used viral model for MS. Although the precise pathophysiology is unknown, altered fecal bacterial populations were associated with distinct immune gene expressions in the CNS. We aimed to determine the role of gut microbiota in TMEV infection by administering an antibiotic cocktail in drinking water before (prophylactic administration) or after (therapeutic administration) TMEV infection. The antibiotic administration reduced total eubacteria, including the phyla *Bacillota* and *Bacteroidota*, but increased the phylum *Pseudomonadata* in feces. Prophylactic administration did not alter TMEV-induced inflammatory demyelination clinically or histologically, without changes in anti-viral IgG1/IgG2c levels or lymphoproliferative responses; therapeutic administration temporarily suppressed the neurological signs. Although antibiotic treatment had minimal effects on TMEV infection, adding metronidazole and ampicillin in drinking water substantially reduced water intake in the antibiotic group of mice, resulting in significant body weight loss. Since dehydration and stress could affect immune responses and gut microbiota, caution should be exercised when planning or evaluating the oral antibiotic cocktail treatment in experimental animals.

## 1. Introduction

Gut microbiota has been shown to play multiple physiological roles, maintaining gastrointestinal homeostasis, protecting against pathogenic microbes, and modulating mucosal and systemic immunities [1,2,3,4]. On the other hand, some pathogens, including viruses, have been reported to utilize the microbiota for their own benefit [5]. For example, the mouse mammary tumor virus could use lipopolysaccharides produced by commensal microbiota for their transmission [6] and poliovirus can use the gut microbiota to enhance infection, resulting in more severe clinical outcomes [7]. The gut microbiota has also been demonstrated to affect the balance between pro- and anti-inflammatory immune responses and play a crucial role in experimental microbial infections and disease models [8,9]. Clinically, gut microbiota dysbiosis has been linked to various immune-mediated diseases, such as inflammatory bowel disease and multiple sclerosis (MS) [10,11,12].

MS is one of the most common immune-mediated diseases of the central nervous system (CNS), characterized by inflammatory demyelination and axonal degeneration in the white matter of the CNS [13,14,15]. Although the precise pathomechanism of MS is still unknown, two primary etiologies have been proposed to trigger MS: autoimmune responses against myelin sheaths (autoimmune theory) and viral infection (viral theory) [16]. The autoimmune theory of MS has been supported by clinical findings, including immune cell infiltration in the demyelinating lesions, associations with major histocompatibility complex (MHC) class II, and the efficacy of immunomodulatory therapy [17,18]. An autoimmune model of MS, experimental autoimmune encephalomyelitis (EAE), has also supported the autoimmune theory, where EAE can be induced by sensitization with CNS antigens, including myelin oligodendrocyte glycoprotein (MOG) and myelin proteolipid protein (PLP) [13,19,20].

The viral etiology of MS has also been supported by clinical and experimental findings. Clinically, several studies have reported that MS patients had higher anti-human herpesvirus 6 (HHV-6) and Epstein–Barr virus (EBV) antibody titers than the healthy controls and that some viruses, including HHV-6, have been isolated from the brain of MS patients [21,22,23,24,25]. Experimentally, several viruses have been demonstrated as inducing MS-like diseases in animals, and Theiler’s murine encephalomyelitis virus (TMEV) has been most widely used as a viral model of MS [26,27,28]. In TMEV infection, the intracerebral inoculation of TMEV in mice resulted in the induction of anti-viral humoral and cellular immune responses that can contribute to not only viral clearance but also immune-mediated damage (immunopathology) of the white matter of the spinal cord [29,30]. In addition, TMEV can persistently infect myelin-forming cells, oligodendrocytes, contributing to demyelination (viral pathology) [30,31].

The gut microbiota has been associated with the progression and severity of human MS and its EAE models [32,33,34,35]. For example, considerable changes have been observed in the gut microbiota of MS patients, compared with the controls [36,37,38]. Germ-free mice developed milder EAE with lower pro-inflammatory interferon (IFN)-γ and interleukin (IL)-17 productions and higher anti-inflammatory regulatory T (Treg) cell responses than conventionally colonized mice [39]. In TMEV infection, gut bacterial populations have been shown to change during the time course [40,41] and were associated with distinct immune gene expressions in the CNS, including antibodies, T cell receptors, and MHC molecules [40]. This suggested that in TMEV infection, gut microbial changes could affect anti-viral immune responses, contributing to the development of MS-like lesions in the CNS. Alternatively, activated immune responses induced by TMEV infection may alter the gut microbiota compositions. Previously, we demonstrated that the activation of immune responses outside the gut could result in gut microbiota changes without induction of any clinical abnormalities [42].

The depletion and modulation of gut bacteria by antibiotic administrations provided insights into the physiological and pathogenic roles of gut microbiota in health and diseases. In human MS and its animal models, however, the effects of antibiotics remained controversial. For example, although Alonso et al. reported that penicillin treatment decreased the risk of MS [43], other research groups reported that antibiotic treatment was a risk factor for MS [44,45]. Antibiotic treatment cases in humans may be inappropriate for assessing the role of gut microbiota in diseases, since antibiotic treatment can (1) affect bacterial populations in not only the gut but also other organs and (2) prevent and suppress microbial infections, which have been reported to exacerbate MS [43]. On the other hand, in experimental animals, the oral administration of non-absorbing antibiotics affecting only the bacteria in the gastrointestinal tract has been used to investigate the role of gut microbiota. In EAE models, oral antibiotic treatment has been reported to suppress, exacerbate, or not affect EAE severities [46,47,48,49,50]. The differences in the antibiotic efficacies of EAE could be due to several factors, including the animal species and strains, treatment timing and duration, and antibiotic compositions. Among the oral antibiotic treatment methods, administering antibiotic cocktails in drinking water has been reported to achieve the most comprehensive bacterial depletion with the least stress in experimental animals [51].

In this study, we aimed to clarify the role of gut microbiota in TMEV infection, a viral model of MS, by depleting gut microbiota. We administered an antibiotic cocktail to mice in drinking water before (prophylactic treatment) or after (therapeutic treatment) TMEV infection (Table 1). The prophylactic antibiotic treatment did not alter TMEV-induced inflammatory demyelination clinically or histologically with no changes in anti-viral immune responses, although the therapeutic treatment suppressed the neurological signs temporarily. Thus, these results suggest that the gut microbiota seemed to play only a minor role in TMEV infection. On the other hand, when we assessed the overall health of experimental mice during the antibiotic treatment, the mice had significant body weight loss with severe dehydration. Since dehydration and stress have been demonstrated to affect immune responses, gut microbiota, and MS models, caution should be exercised in planning or evaluating the oral antibiotic cocktail treatment in experimental animals.

## 2. Materials and Methods

### 2.1. Animals

We purchased 4-week-old SJL/J mice from the Jackson Laboratory, Japan, Inc. (Yokohama, Japan). Mice were maintained under specific pathogen-free conditions in the animal care facility at Kindai University Faculty of Medicine (Osaka, Japan). The Institutional Animal Core and Use Committee of Kindai University Faculty of Medicine approved all experimental procedures performed according to the National Institutes of Health (NIH) outline criteria [52].

### 2.2. TMEV Infection, Antibiotic Treatment, and Sample Collection

On day 0, we inoculated 7–8 week-old mice intracerebrally (i.c.) with 2 × 10^5^ plaque-forming units (PFUs) of the Daniels (DA) strain of TMEV [53]. Mice received an antibiotic cocktail in tap water containing ampicillin (FUJIFILM Wako Pure Chemical Corporation, Osaka, Japan), metronidazole (FUJIFILM Wako Pure Chemical Corporation), neomycin sulfate (Thermo Fisher Scientific Inc., Waltham, MA, USA), and vancomycin (Mylan pharmaceutical Co, Osaka, Japan) [54], either by oral gavage (OG) or in a water bottle (WB). By OG, the mice received a 200 μL antibiotic cocktail solution with a final concentration of 1 g/L of ampicillin, 1 g/L of metronidazole, 1 g/L of neomycin sulfate, and 0.5 g/L of vancomycin per day; control mice received tap water. The WB contained the antibiotic cocktail composed of ampicillin (1 g/L), metronidazole (1 g/L), neomycin sulfate (1 g/L), and vancomycin (0.5 g/L). We conducted two independent experiments (Table 1). In experiment (Exp.) 1, mice received the antibiotic cocktail by OG (days −25 to −21, and days 1 to 14) and in a WB (days −20 to 0). In Exp. 2, mice received the antibiotic cocktail by OG (days 14 to 18 and days 32 to 38) and in a WB (days 19 to 31). To avoid dehydration of the antibiotic group, we injected lactated Ringer’s solution (Solulact^®^, Terumo Corporation, Tokyo, Japan) into mice with 400 μL subcutaneously and 400 μL intraperitoneally (Exp. 1, from days −13 to −9; and Exp. 2, from days 21 to 28).

We monitored mice daily for their body weight changes and clinical signs during the 5-month observation period. We evaluated the clinical signs by the righting reflexes; the proximal end of the mouse’s tail was gripped and twisted to the right and then left sides. The impaired righting reflex was scored as follows: 0, no sign, a healthy mouse resisted being turned over; 1, the mouse was flipped onto its back but immediately righted itself on one side; 1.5, the mouse was flipped onto its back but immediately righted itself on both sides; 2, the mouse righted itself in 1 to 5 seconds (s); 3, righting took more than 5 s; and 4, the mouse could not right itself [55]. We collected fecal samples on days −1, 14, 27, 87, and 147 in Exp. 1. Fecal samples were frozen in liquid nitrogen and stored at −80 °C until further analysis. At 5 months post-infection (p.i.), we killed mice with isoflurane (FUJIFILM Wako Pure Chemical Corporation), collected blood from the heart, and harvested the spleen. We also collected the feces to examine the bacterial abundance in the gut. Then, we perfused mice with phosphate-buffered saline (PBS), followed by a 4% paraformaldehyde (PFA, FUJIFILM Wako Pure Chemical Corporation) solution in PBS, and harvested the spinal cord.

### 2.3. DNA Extraction and Real-Time PCR

We extracted DNA from feces using the QIAamp^®^ Fast DNA Stool Mini Kit (Qiagen, Germantown, MD, USA), according to the manufacturer’s instructions [40]. Real-time PCR was performed on a StepOnePlus system (Thermo Fisher Scientific Inc.). Each sample was run in duplicates, using an optical 96-well plate (INA OPTIKA CO., LTD, Tokyo, Japan). The PCR master mix consisted of 5 μL of SYBR + ROX mix (Toyobo Co., Ltd., Osaka, Japan), 0.3 μL (10 μM) of each primer set (Eurofins Genomics K.K. Tokyo, Japan) shown in Table 2 [56,57,58,59,60,61], 0.4 μL of nuclease-free water, and 4 ng of genomic DNA. The reaction was performed under the following conditions: denaturation at 95 °C for 10 minutes (min), followed by 40 cycles of one-step thermal cycling, consisting of 15 s at 95 °C and 1 min at 60 °C, in a 96-well plate. The relative abundance of bacterial DNA was shown as an arbitrary unit compared with the control Ct values on day −1, using the following formula: 2^−ΔCt^ (ΔCt = Ct value at a time point − Ct value of control group on day −1).

### 2.4. Neuropathology

The spinal cord was divided into 10 to 12 transversal segments and embedded in paraffin. We made 4 μm thick sections using the HM 325 Rotary Microtome (Thermo Fisher Scientific Inc.) [62]. For myelin visualization, we stained the spinal cord sections with Luxol fast blue (Solvent Blue 38, MP Biomedicals, LLC, Irvine, CA, USA) and evaluated neuropathology as described previously [63]. We divided each spinal cord section into four quadrants: the ventral funiculus, dorsal funiculus, and two lateral funiculi. Any quadrant containing meningitis, perivascular cuffing (inflammation), or demyelination was given a score of 1 in that pathological class. The total number of positive quadrants for each pathological class was determined and then divided by the total number of quadrants present on the slide and multiplied by 100 to obtain the percentage of involvement for each pathological class. An overall pathology score was also determined by recording a positive score if any pathology was observed in the quadrant and presented as the percentage of involvement [64].

We visualized the TMEV-antigen by immunohistochemistry using an anti-TMEV antibody [65], Histofine MAX-PO kit (Nichirei Biosciences Inc., Tokyo, Japan), and 3,3′-diaminobenzidine tetrahydrochloride (DAB, FUJIFILM Wako Pure Chemical Corporation). To quantify TMEV antigen-positive (^+^) cells, each spinal cord segment was divided into four funiculi: the ventral funiculus, the dorsal funiculus, and the two lateral funiculi. TMEV antigen^+^ cells per funiculus were counted under a light microscope using a 10× objective lens. The overall viral antigen^+^ cell number per funiculus was also determined by counting all viral antigen^+^ cells in the spinal cord section and then dividing by the total number of funiculi in the spinal cord section. We used 10 to 12 transverse spinal cord segments per mouse of five to eight spinal cords per group in Exp. 1.

### 2.5. Anti-TMEV Antibody Enzyme-Linked Immunosorbent Assays (ELISAs) and Lymphoproliferative Assay

To quantify anti-TMEV antibodies, we obtained the sera from the blood samples by centrifugation at 2775× *g* at 4 °C for 20 min and conducted ELISAs as described previously [62]. We coated a 96-well flat-bottom Nunc-Immuno plate (Thermo Fisher Scientific Inc.) with 10 μg/mL of TMEV antigens. We blocked the plate with an assay diluent consisting of 10% fetal bovine serum (FBS, Sigma-Aldrich Japan K.K., Tokyo, Japan) and 0.2% Tween 20 (FUJIFILM Wako Pure Chemical Corporation) in PBS. We diluted the serum samples with the assay diluent by serial two-fold dilutions from 2^7^ to 2^28^, added diluted samples to the plates, and incubated the plates for 75 min at room temperature (RT) [62]. The plates were washed with a washing buffer containing 0.2% Tween 20 in PBS. Then, we added horseradish peroxidase (HRP)-conjugated anti-mouse immunoglobulin (Ig)G (H+L) (2000-fold dilution, Thermo Fisher Scientific Inc.), anti-mouse IgG1 (4000-fold dilution, Thermo Fisher Scientific Inc.), or anti-mouse IgG2c (4000-fold dilution, Southern Biotechnology Associates, Inc., Birmingham, AL, USA) antibody to the plates and incubated for 90 min at RT. The immunoreactive reaction was developed using the BD OptEIATM TMB Substrate Reagent Set (BD Biosciences, San Jose, CA, USA), according to the manufacturer’s instructions, and stopped with a 2N sulfuric acid (H_2_SO_4_, Wako Pure Chemical Industries) solution. The absorbance was measured at 450 nm on the Synergy H1 Hybrid Multi-Mode Microplate Reader (Agilent Technologies, Inc., Santa Clara, CA, USA). The anti-TMEV antibody titers were determined as the highest reciprocal of the dilution with an absorbance higher than the average plus two standard deviations of naïve serum samples at a dilution of 2^7^-fold.

To determine anti-TMEV cellular immune responses, we harvested and mashed the spleen on a metal mesh with 50 μm pores using the plunger of a 5-mL syringe [62]. The splenic mononuclear cells (MNCs) were isolated using Histopaque^®^-1083 (Sigma-Aldrich Japan K.K). The MNCs were cultured in RPMI-1640 medium (Sigma-Aldrich Japan K.K) supplemented with 10% FBS (Sigma-Aldrich, Co), 2 mM L-glutamine (Sigma-Aldrich Japan K.K), and 50 mM β-mercaptoethanol (FUJIFILM Wako Pure Chemical Corporation) at 2 × 10^5^ cells/well in a 96-well plate (Sumitomo Bakelite Co., Ltd., Tokyo, Japan). We incubated the MNCs with or without TMEV at a multiplicity of infection (MOI) 5 at 37 °C with 5% CO_2_ for 5 days. To quantify the levels of lymphoproliferative responses to TMEV, we added 3 μL/well of a cell-counting kit-8 (CCK-8) solution (Dojindo Laboratories, Kumamoto, Japan) and incubated for the last 24 hours. We conducted the cell culture in triplicate and measured the absorbance at 450 nm using the Synergy H1 Hybrid Multi-Mode Microplate Reader. The results were expressed as stimulation indexes: (mean absorbance of wells stimulated with TMEV)/(mean absorbance of wells without stimulation).

### 2.6. Statistical Analysis

For statistical analysis, we used Origin Pro 2025 (Origin Lab Corporation, Northampton, MA). We conducted the Mann–Whitney *U* test, Student’s *t* test for comparison of two groups, and the Kruskal–Wallis test with Dunn’s post hoc test and analysis of variance (ANOVA) with Fisher’s post hoc LSD test for comparison of three or more groups for nonparametric and parametric data. *p* < 0.05 was considered a significant difference between the groups.

## 3. Results

### 3.1. Effects of Antibiotic Treatment on Body Weights and Clinical Signs in TMEV Infection

**Figure 1:**
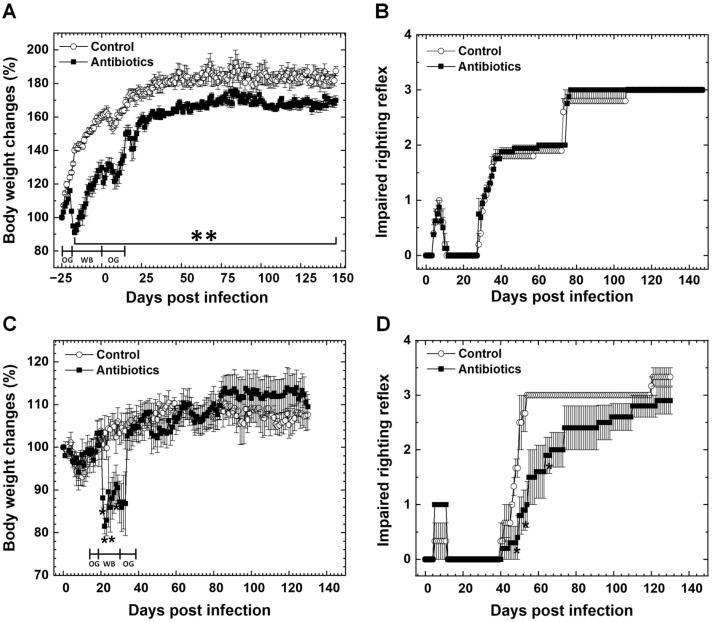
Effect of antibiotic treatment on Theiler’s murine encephalomyelitis virus (TMEV)-infection. (**A**,**B**) In experiment (Exp.) 1, both antibiotic and control groups were inoculated with TMEV on day 0. Mice received the antibiotics (Antibiotics, ■) or tap water (Control, ○) by oral gavage (OG, days −25 to −21, and days 1 to 14) and in a water bottle (WB, days −20 to 0). (**A**) The antibiotic group lost their body weight significantly than the control group when the antibiotics were given in a WB (** *p* < 0.01, Student’s *t* test). (**B**) We assessed neurological signs by impaired righting reflex scores and found no statistical difference between the antibiotic and control groups (Mann–Whitney *U* test). (**C**,**D**) In Exp. 2, on day 14, the mice received either an antibiotic cocktail or tap water by OG for 5 days, followed by administration of the antibiotic cocktail or tap water in a WB for 14 days from days 19 to 31, and by OG again for 1 week from days 32 to 38. (**C**) From days 22 to 28, the antibiotic group had significant loss of body weight, compared with the control group (* *p* < 0.05). (**D**) The antibiotic group had less severe neurological disabilities than the control group (* *p* < 0.05) from days 48 to 67, but later caught up with the clinical score, showing no significant differences from day 68 throughout the disease course. Results are the mean ± standard error of the mean (SEM) of three to eight mice per group.

We inoculated mice with TMEV i.c. on day 0. To determine whether modulation of gut microbiota could affect the clinical course of TMEV infection, we orally administered mice with an antibiotic cocktail or tap water daily from day −25 to day 14 (Exp. 1). We monitored the body weight of mice and clinical signs for 5 months p.i. In the antibiotic group, mice received the antibiotics by OG (days −25 to −21 and days 1 to 14) and in a WB (days −20 to 0). The antibiotic group avoided drinking the antibiotic cocktail solution in a WB; the mice lost body weight from days −19 to −14, leading to severe dehydration. Although the mice in the antibiotic group gained body weight later, they did not catch up with the body weight of the control mice during the observation period (Figure 1A).

We assessed neurological signs by impaired righting reflex scores and found both the antibiotic and control groups had mild righting reflex impairment 1-week p.i. (acute phase) and recovered completely. Then, mice developed righting reflex impairment, which reflected the onset of demyelinating disease in the spinal cord from 1-month p.i. (chronic phase) and exhibited the neurological disabilities progressively. There were no significant differences in the impaired righting reflex scores between the two groups (Figure 1B).

In Exp. 2, TMEV-infected mice were treated with an antibiotic cocktail from days 14 to 38: days 14 to 18 and days 32 to 38 by OG and days 19 to 31 in a WB. From days 22 to 28, the antibiotic group showed a significant decrease in body weight, compared with the control group (Figure 1C). This was associated with severe dehydration; mice avoided drinking an antibiotic cocktail solution in a WB. In the early chronic phase, the impaired righting reflex scores of the antibiotic group were lower than the control group (days 48 to 67) but later caught up with righting reflex impairment of the control mice during the observation period (Figure 1D). 

### 3.2. Antibiotic Treatment and Neuropathology of TMEV Infection

**Figure 2:**
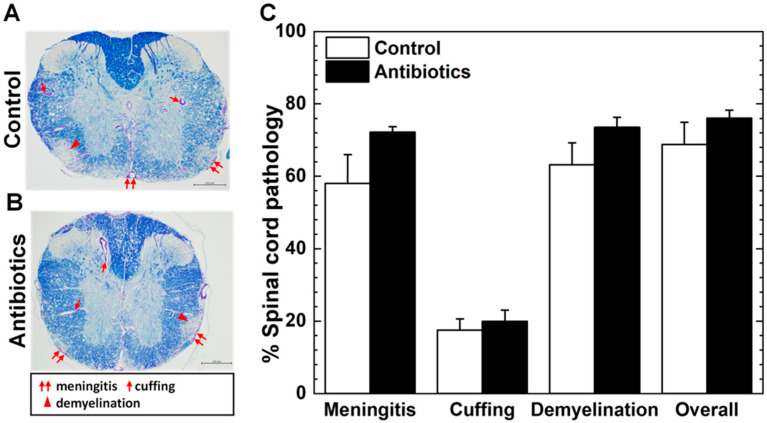
Spinal cord pathology of TMEV-infected mice. (**A**,**B**) In the antibiotic and control groups, we found similar distribution and severities of meningitis (paired arrows), perivascular cuffing (arrows), and demyelination (arrowheads). Scale bar: 200 μm. (**C**) We quantified spinal cord pathology using the spinal cord pathology scoring system. There were no significant differences in the levels of meningitis, perivascular cuffing, demyelination, or overall pathology between the antibiotic (closed bar) and control (open bar) groups. Results are shown as the mean + SEM of five to eight mice per group in Exp. 1.

During the chronic phase, TMEV has been shown to induce inflammatory demyelinating lesions in the white matter of the spinal cord, similar to human MS. We stained the spinal cord sections with Luxol fast blue for myelin visualization and compared the neuropathology between the antibiotic and control groups. In both the antibiotic and control groups in Exp. 1, we observed no significant differences in the distribution and severities of meningitis, perivascular cuffing (inflammation), and demyelination in the spinal cord (Figure 2A–C). Similarly, in Exp. 2, we found no significant differences in neuropathology scores between the two groups (Supplementary Figure S1A,B), although the antibiotic group had lower levels of meningitis, inflammation, and demyelination in the spinal cord than the control group (Supplementary Figure S1C).

### 3.3. Antibiotic Treatment and Viral Persistence

**Figure 3:**
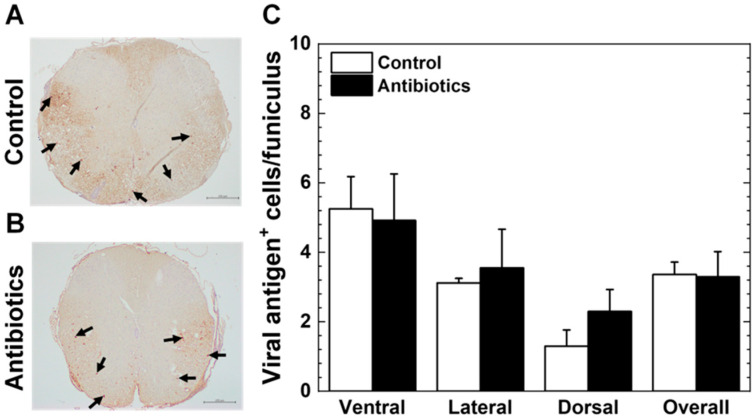
Immunohistochemistry against viral antigens of the spinal cord 5 months post infection (p.i.). (**A**,**B**) We found similar number and distribution of viral antigen^+^ cells (arrows) in the spinal cord of the antibiotic and control groups. Scale bar: 200 μm. (**C**) We quantified viral antigen^+^ cells and found comparable numbers of viral antigen^+^ cells in the ventral and lateral funiculi of the spinal cord between the antibiotic (closed bar) and control (open bar) groups. A small number of viral antigen^+^ cells were detected in the dorsal funiculus of both groups. The mean number of viral antigen^+^ cells per funiculus (Overall) was also determined by counting all viral antigen^+^ cells and spinal cord funiculi present on the slide. There were no significant differences in the numbers of viral antigen^+^ cells/funiculus in the ventral, lateral, dorsal funiculi, or overall, between the antibiotic and control groups. Results are the mean + SEM of five to eight mice per group and 10–12 spinal cord transverse sections per mouse in Exp. 1.

In TMEV infection, the presence of viral antigen^+^ cells, i.e., viral persistence, in the white matter of the spinal cord has been demonstrated to be necessary for the induction of inflammatory demyelination. We compared the numbers of viral antigen^+^ cells between the antibiotic and control groups by immunohistochemistry with anti-TMEV antibody. In both groups, viral antigen^+^ cells were mainly detected in the white matter of the ventral and lateral funiculi of the spinal cord (Figure 3A,B, Supplementary Figure S2A,B), and their numbers were comparable between the two groups. Although a small number of viral antigen^+^ cells were also detected in the dorsal funiculus of the spinal cord in both groups, there were no significant differences between the groups (Figure 3C, Supplementary Figure S2C).

### 3.4. Anti-Viral Antibody Isotypes and Lymphoproliferative Responses

**Figure 4:**
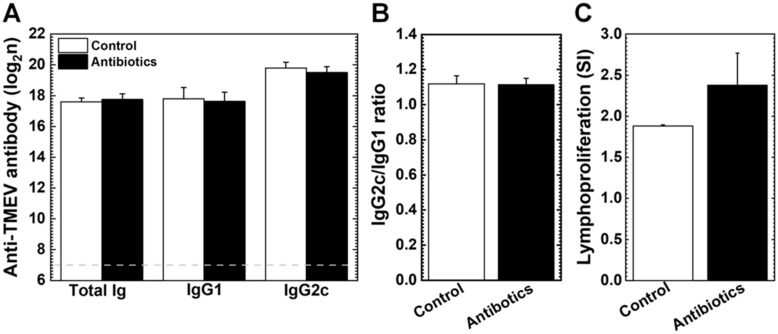
Humoral and cellular immune responses against TMEV. We harvested sera and spleens from the antibiotic (closed bar) and control (open bar) groups at 5 months p.i. (**A**) We quantified anti-TMEV antibodies: total immunoglobulin (Ig), IgG1, and IgG2c. We did not find significant differences in the levels of anti-TMEV antibodies. (**B**) In IgG2c versus IgG1 ratios, which reflected Th1/Th2 balance, there were no significant differences between the two groups. The results are the mean + SEM of five to eight mice per group of Exp. 1. (**C**) Lymphoproliferative responses to TMEV were expressed as stimulation indexes (SI). There was no significant difference in the lymphoproliferative responses between the two groups. Each group was composed of three pools, and each pool included spleens from two to three mice.

To determine whether the antibiotic treatment could affect humoral and cellular immune responses in TMEV infection, we collected the sera and splenic MNCs from the antibiotic and control groups at 5 months p.i. Using ELISAs, we found that the antibiotic treatment did not significantly alter anti-TMEV total antibody, IgG1, and IgG2c isotype antibody levels (Figure 4A, Supplementary Figure S3A). We calculated the ratio of IgG2c [reflecting T helper (Th)1 cytokine response] versus IgG1 (reflecting Th2 cytokine response) and found no significant differences between the two groups (Figure 4B, Supplementary Figure S3B). We also compared anti-TMEV cellular immune responses by lymphoproliferative assays using splenic MNCs. We did not find significant differences in TMEV-specific lymphoproliferative responses between the two groups (Figure 4C, Supplementary Figure S3C).

### 3.5. Effects of Antibiotic Treatment on Gut Microbiota in TMEV Infection

**Figure 5:**
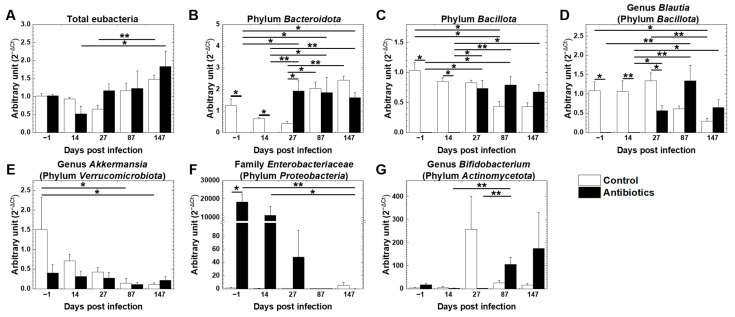
Time course analyses of gut microbiota in TMEV-infected mice received an antibiotic cocktail (Antibiotics, closed bar) or tap water (Control, open bar) from days −25 to 14: (**A**) total eubacteria, (**B**) the phylum *Bacteroidota*, (**C**) the phylum *Bacillota*, (**D**) the genus *Blautia*, (**E**) the genus *Akkermansia*, (**F**) the family *Enterobacteriaceae*, and (**G**) the genus *Bifidobacterium*. Results are shown as the mean + SEM of five to eight mice per group in Exp. 1 and were expressed by an arbitrary unit calculated by the following formula: 2^−ΔCt^ (ΔCt = Ct value at a time point − Ct value of the control group on day −1). The significance of the difference was calculated by the Mann–Whitney *U* test for two groups and the Kruskal–Wallis test for three or more groups. * p < 0.05, ** p < 0.01.

Using the fecal samples from the antibiotic and control groups, we evaluated the effects of antibiotic treatment on gut microbiota. To determine which bacterial taxa were affected by TMEV infection as well as antibiotic treatment, we conducted real-time PCR for six taxa that could cover most of the gut bacteria detected in murine feces, as we and other groups have reported previously [40,56,57,58,59,60,61]. In the control TMEV-infected group, compared with day −1 (prior to TMEV infection), the levels of total eubacteria and the phylum *Bacteroidota* were temporally decreased on day 27 and increased from day 87 (Figure 5A,B). The phylum *Bacillota* and its genus *Blautia* as well as the genus *Akkermansia* (belonging to the phylum *Verrcomicrobiota*) declined substantially during the chronic phase of infection on days 87 and 147 (Figure 5C–E). Low levels of bacteria were detected in the family *Enterobacteriaceae*, representative of the family in the phylum *Pseudomonadota* in the gut, and the genus *Bifidobacterium*, representative of the genus in the phylum *Actinomycetota* in the gut (with the exception of day 27) throughout the disease course (Figure 5F,G).

In the group treated with antibiotics from day −25 to day 14, the relative abundance of total eubacteria was reduced on day 14 but increased on day 27 (Figure 5A). Thus, the antibiotic treatment decreased the bacteria temporarily but did not deplete the bacteria completely; incomplete depletion of gut bacteria by antibiotic cocktail treatment was consistent with previous reports [66,67,68]. The phylum *Bacillota* and its genus *Blautia* and the phylum *Bacteroidota* were decreased on days −1 and 14 but recovered on day 27 (Figure 5B–D). In contrast, the family *Enterobacteriaceae* increased significantly on days −1 and 14 but decreased on days 87 and 147, compared with the control group (Figure 5F). Thus, overall, the antibiotic treatment seemed to kill mainly the bacteria belonging to the phyla *Bacillota* and *Bacteroidota*, resulting in the increase in the family *Enterobacteriaceae*. On the other hand, the antibiotic treatment decreased the genus *Akkermansia* throughout the disease course (Figure 5E). The genus *Bifidobacterium* was significantly increased in the antibiotic group on day 87 (Figure 5G).

## 4. Discussion

In this study, we conducted real-time PCR of six bacteria taxa to cover most of the gut bacteria detected in murine feces, as reported previously [60,61]. The phyla *Bacillota* (formerly *Firmicutes*) and *Bacteroidota* (formerly *Bacteroidetes*) have been reported as anti-inflammatory and pro-inflammatory taxa, respectively [69]. Although the *Bacillota*/*Bacteroidota* ratios were associated with the activities of various diseases, including inflammatory bowel disease and obesity [69,70,71], the association of *Bacillota*/*Bacteroidota* ratios with disease activities of MS and its animal models was not consistent among the previous reports [36,72,73]. In TMEV infection, we previously demonstrated that the phyla *Bacillota* and *Bacteroidota* were dominant taxa, and their relative abundances were not changed during the first 1 month p.i. [40]. Similarly, in the current study, although both phyla declined until day 27, the *Bacillota*/*Bacteroidota* ratios seemed unaltered. We did not detect the family *Enterobacteriaceae* and the genus *Bifidobacterium*, which was consistent with our previous report. From day 87, since the phyla *Bacillota* and *Bacteroidota* were decreased and increased, respectively, the ratio of the phyla *Bacillota*/*Bacteroidota* was reduced, compared with the early stage of infection. Mestre et al. [66] reported that in TMEV infection, the antibiotic treatment decreased the phyla *Bacillota* and *Bacteroidota* and increased the phyla *Pseudomonadota* (formerly *Proteobacteria*) and *Actinomycetota* (formerly *Actinobacteria*), which was consistent with our current results.

At the family/genus levels, although altered bacterial abundance in several bacterial taxa, including the genera *Akkermansia*, *Blautia*, and *Bifidobacterium*, has been reported in MS and its animal models, the findings were often inconsistent among the reports. For example, Zhou et al. [74] and Cox et al. [75] reported increased *Akkermansia* and *Bifidobacterium* and decreased *Blautia* in MS patients compared with healthy controls. On the other hand, Thirion et al. [76] reported an increase in the genus *Blautia*, but not *Akkermansia* in MS; Ghimire et al. [77] suggested that increased *Blautia* and a lower *Bifidobacterium*/*Akkermansia* ratio in MS and its EAE model. In TMEV infection, Carrilo-Salinas et al. [54] reported decreased *Akkermansia* in untreated TMEV-infected mice as well as increased *Bifidobacterium* after antibiotic treatment. In our previous and current TMEV studies, the changes in the genera *Akkermansia*, *Blautia*, and *Bifidobacterium* with or without antibiotic treatment were irrelevant to the disease activity [40].

Gut microbiota has been shown to facilitate or inhibit viral infections by two mechanisms: modulating immune responses and interacting with the viruses [78]. Gut microbiota can contribute to protection from various viral infections in the gut and other organs by regulating anti-viral immune responses [79]. For example, the administration of an antibiotic cocktail solution to C57BL/6 mice induced severe dysbiosis by depleting gut microbiota, resulting in severe respiratory viral infections [80]. On the other hand, enteric viruses, including poliovirus that belongs to the family *Picornaviridae*, have been shown to use gut microbiota to advance infection: the gut microbiota depletion by antibiotic cocktails diminished viral replication [7]. Thus, we hypothesized that the depletion of gut microbiota by antibiotic treatment could alter the anti-viral immune responses and viral replication in TMEV infection. However, this was not the case; we found similar levels of viral antigen^+^ cells as well as anti-viral humoral and cellular immune responses between the antibiotic and control groups (Figure 3, Figure 4 and Supplementary Figure S3).

Antibiotics can kill commensal microbes in the gut besides pathogenic bacteria, causing gut microbiota dysbiosis [81,82,83]. In the current study, we used an antibiotic cocktail composed of four antibiotics, ampicillin, metronidazole, neomycin sulfate, and vancomycin, to decrease both the aerobic and anaerobic bacteria in the gut. Ampicillin is a penicillin with a broad-spectrum activity against bacteria, including enterococci [80,84]. Metronidazole is converted to nitroso compound by nitroreductase derived from anaerobic bacteria and is effective against protozoa and anaerobic bacteria, including *Bacteroides fragilis* and *Clostridium difficile* [85,86]. Neomycin sulfate is an aminoglycoside that is effective only for the aerobic bacteria [87,88]. Vancomycin is a glycopeptide antibiotic with a broad-spectrum antibiotic activity against Gram-positive bacteria [89]. 

Previously, using the same antibiotic cocktail in drinking water, Gauza’s group reported the effects of prophylactic [54] and therapeutic [66] antibiotic administration in TMEV infection. Consistent with our results, their prophylactic administration did not alter TMEV-induced disease on day 85. On the other hand, our antibiotic treatment did not result in a complete or sustained depletion of gut microbiota (Figure 5); a similar incomplete depletion of gut microbiota by the antibody cocktail treatment has been shown previously [7,90,91,92]. Intriguingly, Gauza’s group reported that their antibiotic treatment depleted the gut bacteria completely on day 14 (the bacteria recovered over time). Furthermore, 40% of antibiotic-treated mice died on day 21 (in a single day); the cause of the death was unknown since no autopsy was conducted. The data on day 14 showed significant decreases in all immune cell types in the brain; viral pathology, but not immunopathology, might cause the sudden death (although neither viral titers nor a possible scenario were provided by the authors) [54]. Since we did not observe any changes in disease activity or sudden death of TMEV-infected mice, the difference in the extent of bacterial depletion between our study vs. Gauza’s study (i.e., incomplete depletion with residual gut bacteria vs. complete depletion) might explain the different outcomes between the two studies. 

As therapeutic administration, Gauza’s group gave the antibiotic cocktail from days 55 to 70, which suppressed motor disability mildly (no differences in a rotarod test, but improved horizontal and vertical activities, compared with the control TMEV-infected group) [66]. The antibiotic group had decreased T-cell infiltration in the CNS; neither viral persistence nor anti-viral immune responses were examined. Although the overall clinical effects on TMEV-induced disease in our current study were similar to those in Gauza’s study, there were differences in the extent of bacterial depletion, mortality, and immune cell infiltration in the CNS between the two studies. In our therapeutic antibiotic administration, we observed the temporal improvement in clinical signs. Although the precise mechanisms were not clarified in our study, one possible mechanism was the temporal modulation of local and systemic immune responses associated with altered gut microbiota, without changing viral burden [79,80]. Alternatively, antibiotic treatment could induce changes in microbial metabolites, such as short-chain fatty acids [66,67], which have been shown to influence host immune responses and CNS immune-mediated diseases [93,94,95].

The differences between our study and Gauza’s study could be due to the inconsistent intake of the antibiotic cocktail in drinking water. Although the antibiotic cocktail in drinking water can reduce gut microbiota efficiently, the unpalatable taste of the antibiotic cocktails, often attributed to metronidazole and ampicillin [96,97], has been reported to prevent water consumption in mice, resulting in dehydration and substantial weight loss [49,67,68,98]. The resistance to the antibiotic cocktails differed among the mouse strains. For example, it has been reported that following antibiotic treatment, SJL/J and NOD mice suffered 20–30% body weight reductions; C57BL/6 mice did not alter body weights [49,50]. Although adding glucose, sucrose, and flavored/acidified water into antibiotic solutions has been reported to increase the palatability of antibiotics in a drinking WB [90,99,100,101], adding 2.5% of sucrose into the antibiotic cocktail solution did not increase water consumption in the current study. Other research groups recommended that if the antibiotic cocktail solution alone was given to mice as drinking water, they would eventually drink it. Again, the strategy did not work in our experiment, and the mice died of dehydration from not drinking the solution. The only method that made mice tolerate taking antibiotic cocktails in a WB was to administer the solution by OG for 5 days before the drinking water administration [92]. Even with this method, by switching the OG into drinking WB, we found significant weight loss in the antibiotic group (Figure 1A,C); we had to inject a large volume of lactated Ringer’s solution into mice, subcutaneously and intraperitoneally.

In our study, there were two limitations. One of the limitations was the presence of several confounding factors associated with the oral antibiotic cocktail administration. As described above, the mice in the antibiotic group suffered from dehydration. Dehydration in the antibiotic group was inferred from the clinical signs, including recessed eyes, fuzzy facial fur, and skin tenting by pinching the skin over the shoulder blades [102]. Previous studies have also shown that antibiotics in drinking water caused dehydration and weight loss in mice [67,68]. Dehydration impaired the mucosal barrier function and augmented pro-inflammatory cytokine production, affecting disease outcomes in CNS infections [103,104]. Dehydration has also been reported to induce significant changes in microbiota, specifically changes in several genera, which could increase the risk of inflammatory disorder with abnormal brain function [105]. Although weight loss in the antibiotic group was likely due to dehydration, we did not quantify water and food intake, using a metabolic cage. If present, food intake loss, i.e., calorie restriction/fasting, could also change the gut microbiota, immune response, and stress levels [106]. In addition, administration by OG allowed a higher and more accurate dose of antibiotics compared with WB administration; the OG administration was more labor-intensive and stressful for mice, particularly when repeated several times, as reported in microbiota depletion experiments [107,108,109,110]. Both dehydration and OG likely caused stress in the antibiotic group. Antibiotic cocktail administration has been demonstrated to induce depression in experimental mice; depression can also be regarded as stress. Stress can affect the host immune responses and alter the gut microbiota [33,111,112,113], increasing the susceptibility to inflammatory or autoimmune diseases [114]. Stress has been shown to affect disease activities in MS and its EAE models, positively or negatively [115,116,117,118]. Stress can also increase susceptibility to microbial infections by suppressing the immune system; various microbial infections have been linked to MS onset and exacerbation [118,119]. In TMEV infection, Welsh et al. conducted a series of experiments and demonstrated that stress suppressed anti-viral immune responses, resulting in higher levels of viral replication and dissemination, and exacerbation of demyelinating disease [119,120,121]. Thus, stress can alter the immune responses and microbiota [114] and may mask or alter the effects of microbiota changes on the TMEV model.

Another limitation was the depth of microbiome analysis. We conducted real-time PCR for six bacterial taxa, covering most gut bacteria detected in murine feces and dominant taxa in TMEV infection. Thus, we could not conduct α-diversity analysis due to the limited number of bacterial phyla and genera. In MS and its animal models, including TMEV infection, α-diversity in gut microbiota has been shown to be irrelevant to disease activities [40,74,76].

In this study, we demonstrated that the oral administration of an antibiotic cocktail in a viral model of MS, TMEV infection, resulted in an alteration of the microbiome without substantial changes in neuropathology or anti-viral immune responses. Although we suggested a minor role of the gut microbiota in TMEV-induced demyelinating disease, the procedure of oral antibiotic administration had several potential confounding factors, such as induction of dehydration and stress, which could affect immune responses and gut microbiota. Thus, caution should be paid to plan or evaluate the oral antibiotic cocktail treatment in experimental animals.

## Figures and Tables

**Table 1:** Antibiotic treatment schedule in TMEV-infected mice.

Exp. No.	Groups	TMEV Infection	Mouse No.	Antibiotic Treatment		
				Oral Gavage	Water Bottle	Oral Gavage
Exp. 1	prophylactic	day 0	8	days −25 to −21	days −20 to 0	days 1 to 14
	control	day 0	5	(−)	(−)	(−)
Exp. 2	therapeutic	day 0	5	days 14 to 18	days 19 to 31	days 32 to 38
	control	day 0	3	(−)	(−)	(−)

Abbreviations: Exp., experiment; No., number; TMEV, Theiler’s murine encephalomyelitis virus.

**Table 2:** List of primer sets used for RT-PCR analyses of gut microbiota.

Target Bacterial Taxa	T_m_ (°C)	Primer Sequence	References
Total eubacteria	68 69	F: CGGYCCAGACTCCTACGGG R: TTACCGAGGCTGCTGGCAC	[56]
Phylum *Bacillota*	65 66	F: GGAGYATGTGGTTTAATTCGAAGCA R: AGCTGACGACAACCATGCAC	[57]
Phylum *Bacteroidota*	62 61	F: GTTTAATTCGATGATACGCGAG R: TTAASCCGACACCTCACGG	[58]
Family *Enterobacteriaceae*	72 61	F: CATTGACGTTACCCGCAGAAGAAGC R: CTCTACGAGACTCAAGCTTGC	[59]
Genus *Bifidobacterium*	59 57	F: TCGCGTCYGGTGTGAAAG R: CCACATCCAGCRTCCAC	[60]
Genus *Akkermansia*	70 68	F: CAGCACGTGAAGGTGGGGAC R: CCTTGCGGTTGGCTTCAGAT	[61]
Genus *Blautia*	71 69	F: TCTGATGTGAAAGGCTGGGGCTTA R: GGCTTAGCCACCCGACACCTA	[61]

Abbreviations: Tm, melting temperature; F, forward primer; R, reverse primer.

## Data Availability

The original contributions presented in this study are included in the article/Supplementary Material. Further inquiries can be directed to the corresponding author.

## Supplementary Materials

The following supporting information can be downloaded at: https://www.mdpi.com/article/10.3390/cells14120871/s1, Figure S1: Effects of antibiotic treatment on neuropathology of TMEV infection in Exp. 2; Figure S2: Viral antigen^+^ cells in TMEV infection in Exp. 2; Figure S3: Anti-viral immune responses in TMEV infection in Exp. 2.
