# Supplementary material for: Gut Microbiota in a Viral Model of Multiple Sclerosis: Modulation and Pitfalls by Oral Antibiotic Treatment
Source: Cells. 2025 Jun 9;14(12):871. doi: 10.3390/cells14120871 (PMC12190502; doi:10.3390/cells14120871)
Supplement: Supplementary file 1 [file cells-14-00871-s001.zip › cells-3597849-supplementary.pdf]

Supplementary Materials

Gut Microbiota in a Viral Model of Multiple Sclerosis: Modulation and Pitfalls by Oral Antibiotic Treatment

Ijaz Ahmad[1†], Seiichi Omura[1†], Sundar Khadka[1,2†], Fumitaka Sato[1], Ah-Mee Park[1,3], Sandesh Rimal[1], and Ikuo Tsunoda[1*]

[1] Department of Microbiology, Kindai University Faculty of Medicine, Osaka, Japan

[2] Department of Medicine, Duke University, Durham, North Carolina, USA

[3] Department of Arts and Science, Kindai University Faculty of Medicine, Osaka, Japan

[†]These authors contributed equally to this work.

***Correspondence:**
Ikuo Tsunoda, M.D., Ph.D.
Department of Microbiology, Kindai University Faculty of Medicine
377-2 Ohnohigashi, Osakasayma, Osaka 589-8511 Japan
E-mail: itsunoda@med.kindai.ac.jp

Table of contents

Supplementary Figure S1. Effects of antibiotic treatment on neuropathology of TMEV infection in Exp. 2...3

Supplementary Figure S2. Viral antigen^{+} cells in TMEV infection in Exp.2……………………………….4

Supplementary Figure S3. Anti-viral immune responses in TMEV infection in Exp. 2………………….…..5

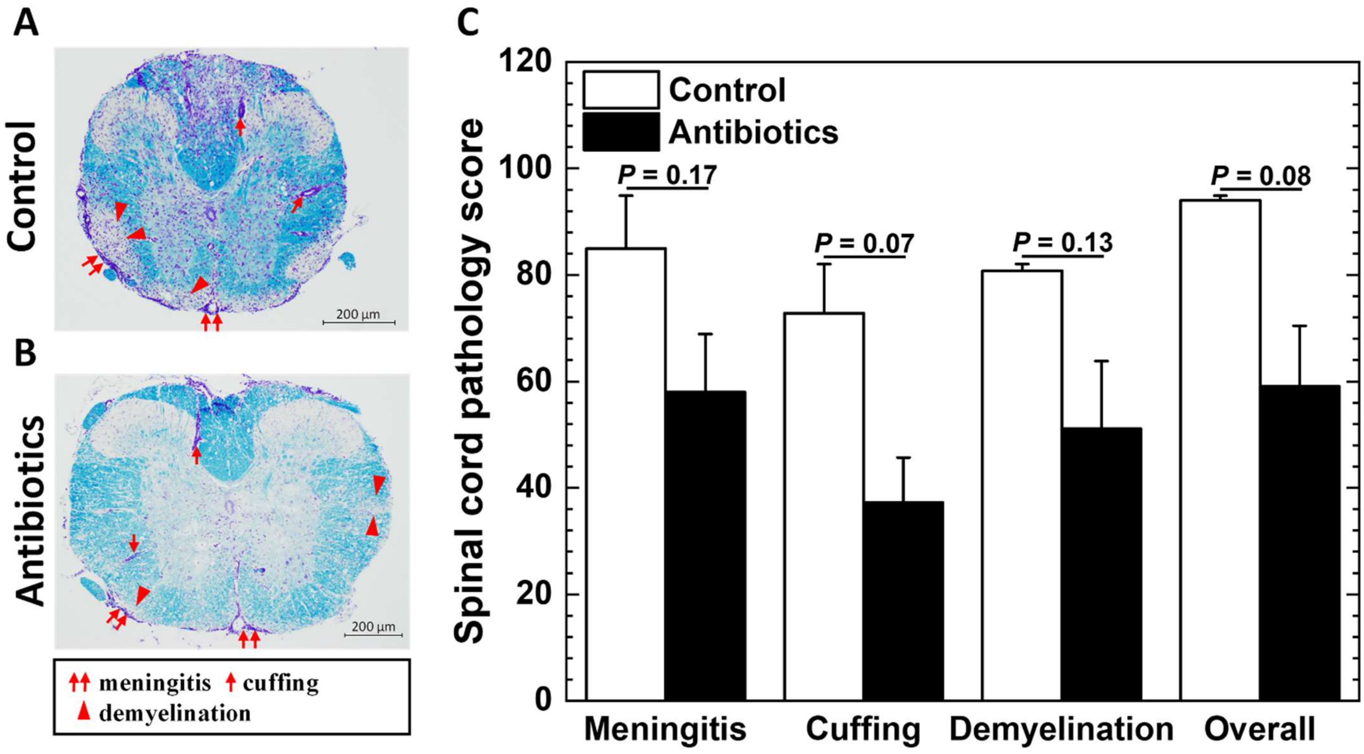

Supplementary Figure S1. Effect of antibiotic treatment on neuropathology of mice infected with Theiler's murine encephalomyelitis virus (TMEV) 5 months post infection (p.i.) in Exp. 2. Mice received an antibiotic cocktail (antibiotic group) or tap water (control group). (**A, B**) Both the antibiotic and control groups developed meningitis (paired arrows), perivascular cuffing (inflammation, arrows), and demyelination (arrowheads). Scale bar: 200 µm. (**C**) We quantified spinal cord pathology scores using a spinal cord pathology scoring system. Although the control group (Control, open bars) had higher pathology scores than the antibiotic group (Antibiotics, closed bars), we did not find significant differences in any pathology categories between the two groups: meningitis, P = 0.17; perivascular cuffing, P = 0.07; demyelination, P = 0.13; and overall pathology, P = 0.08. Results are the mean + standard error of the mean (SEM) of three to five mice per group and 10-12 spinal cord transverse sections per mouse.

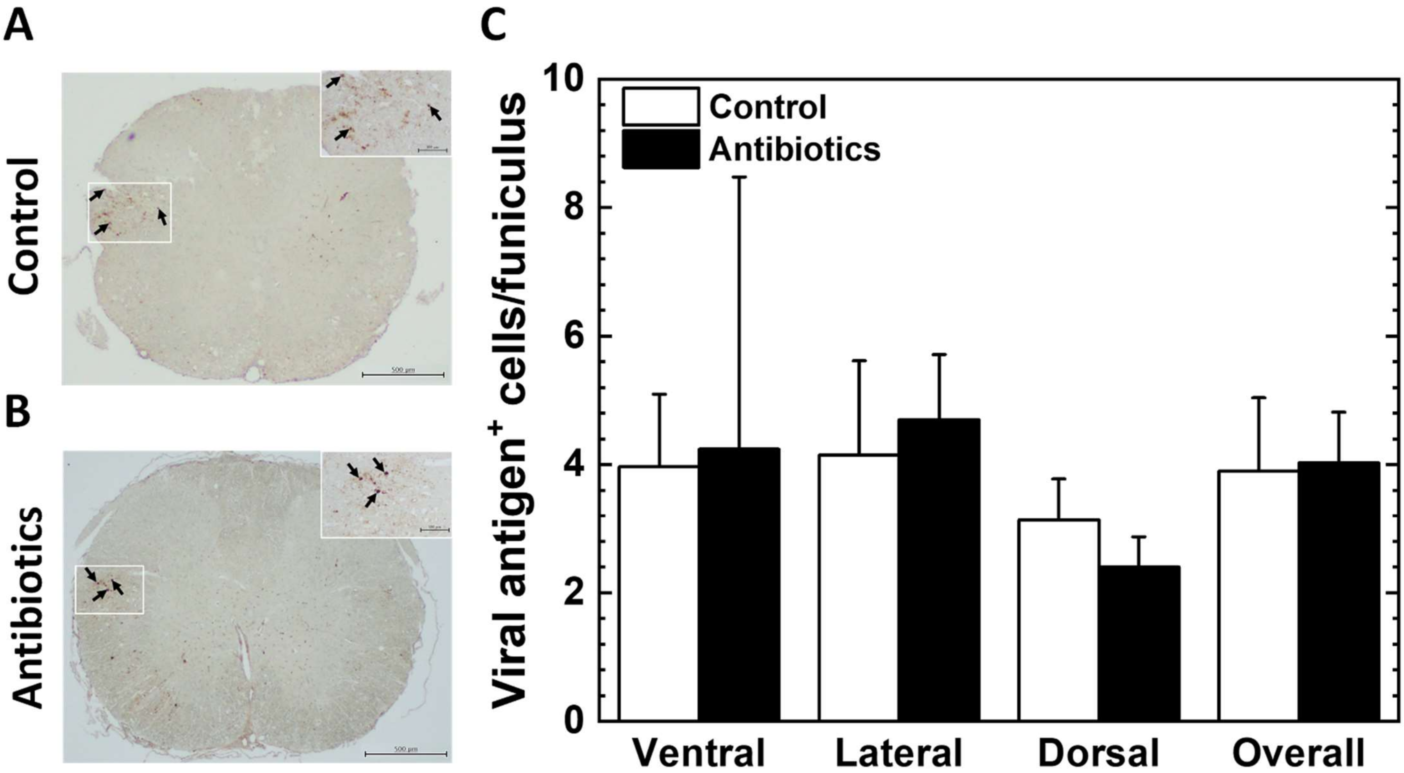

Supplementary Figure S2. Immunohistochemistry against viral antigens of the spinal cord of TMEV-infected mice in Exp.2. (**A, B**) We found similar numbers, distribution, and cell types of viral antigen-positive ($^{+}$) cells (arrows) in the spinal cord of the antibiotic and control groups. Scale bar: 500 μm and inset, 20 μm. (**C**) We quantified viral antigen^{+} cells and found comparable numbers of viral antigen^{+} cells in the ventral and lateral funiculi of the spinal cord between the antibiotic (Antibiotics, closed bar) and control (Control, open bar) groups. The mean number of viral antigen^{+} cells per funiculus (Overall) was also determined by counting all viral antigen^{+} cells and spinal cord funicli present on the slide. The number of viral antigen^{+} cells in the dorsal funiculus was small in both groups. We did not find significant differences in the numbers of viral antigen^{+} cells/funiculus in the ventral, lateral, dorsal funiculi, and overall, between the antibiotic and control groups. Results are the mean + SEM of three to five mice per group and 10–12 spinal cord transverse sections per mouse.

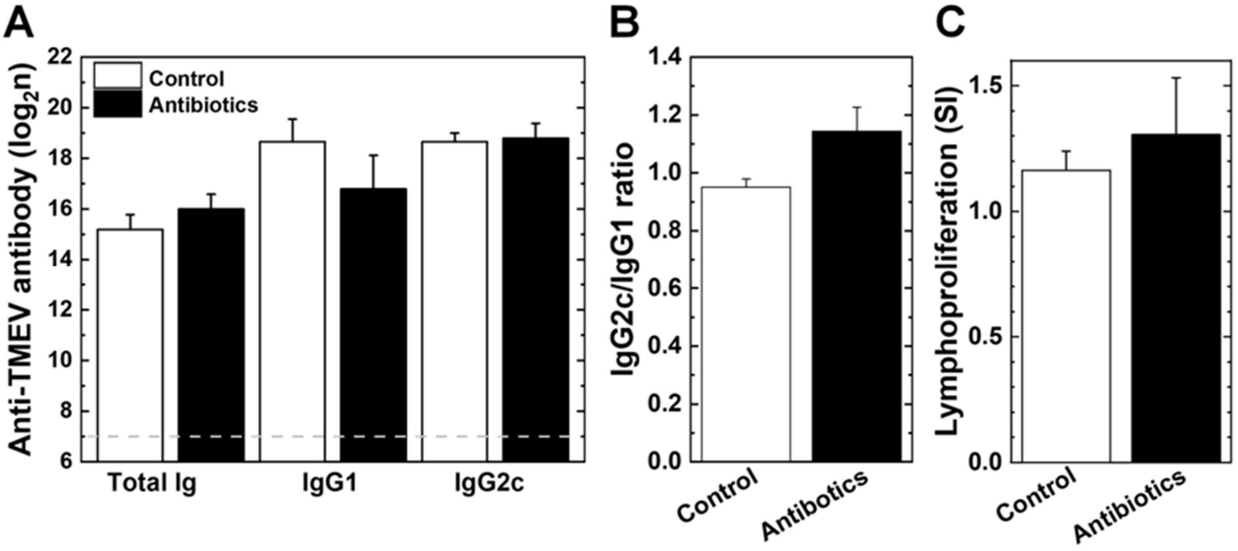

Supplementary Figure S3. Humoral and cellular immune responses against TMEV in Exp. 2. (**A**) Five months p.i., we harvested sera from the antibiotic (Antibiotics, closed bar) and control groups (Control, open bar). We quantified the level of anti-TMEV antibody responses using enzime-linked immunosorbent assays (ELISAs): total immunoglobulin (Ig), IgG1 and IgG2c. Both groups had similar levels of anti-TMEV antibody responses. (**B**) The IgG2c versus IgG1 ratios were similar between the groups. Results are the mean + SEM of three to five mice per group. (**C**) Lymphoproliferative responses to TMEV were quantified by the Cell Counting Kit-8 and were expressed as stimulation indexes (SI). There were no significant differences between the two groups. Each group was composed of two pools, and each pool included the spleen from one to three mice.
